# Repayment Flexibility Can Reduce Financial Stress: A Randomized Control Trial with Microfinance Clients in India

**DOI:** 10.1371/journal.pone.0045679

**Published:** 2012-09-26

**Authors:** Erica Field, Rohini Pande, John Papp, Y. Jeanette Park

**Affiliations:** 1 Department of Economics, Duke University, Durham, North Carolina, United States of America; 2 Harvard Kennedy School, Cambridge, Massachusetts, United States of America; 3 Highbridge Capital Management, New York, New York, United States of America; 4 Harvard Business School, Boston, Massachusetts, United States of America; London School of Economics, United Kingdom

## Abstract

Financial stress is widely believed to cause health problems. However, policies seeking to relieve financial stress by limiting debt levels of poor households may directly worsen their economic well-being. We evaluate an alternative policy – increasing the repayment flexibility of debt contracts. A field experiment randomly assigned microfinance clients to a monthly or a traditional weekly installment schedule (N = 200). We used cell phones to gather survey data on income, expenditure, and financial stress every 48 hours over seven weeks. Clients repaying monthly were 51 percent less likely to report feeling “worried, tense, or anxious” about repaying, were 54 percent more likely to report feeling confident about repaying, and reported spending less time thinking about their loan compared to weekly clients. Monthly clients also reported higher business investment and income, suggesting that the flexibility encouraged them to invest their loans more profitably, which ultimately reduced financial stress.

## Introduction

The success of microfinance – the system that aids poor women in developing countries by offering them small collateral-free loans – was acknowledged internationally in 2006 when Muhammad Yunus and the Grameen Bank won the Nobel Peace Prize [1], [2]. While the microfinance model has increased economic opportunities for the poor, its strict repayment requirement has come under fire in the media after reports of suicides among loan defaulters in the Indian state of Andhra Pradesh in 2010 [3]. A central concern is that the psychological burden of frequent repayment – particularly among poor clients who often lack the financial tools to optimally manage loans – may in many instances offset the positive influence of access to credit, making microfinance borrowers worse off in terms of mental well-being [4].

The typical microfinance borrower faces a very rigid repayment schedule that requires her to make installments on a weekly basis beginning shortly after loan disbursement. While such a contract is believed to be an important component of keeping default at bay [5], frequent repayment also limits clients' ability to deal with short-term shocks to household income and could, therefore, be an important source of anxiety when there is a high degree of income variance. Our study rigorously examines whether a small adjustment in loan structure that reduces repayment rigidity can make it possible for clients to experience the economic benefits of microfinance with minimized financial stress.

Financial stress is well documented in the psychology literature to be an important factor leading to mental health problems [6]–[8], which in turn are among the most important causes of morbidity in the world, and which produce considerable disability in developing countries [9]–[11]. Indicators of poverty and risk for mental disorders are highly correlated in the developing world [12]. Hence, minimizing financial stress is of first-order importance.

Yet, despite the media's portrayal, rigorous evidence that microfinance indebtedness negatively impacts mental health is lacking [13]. Moreover, theory suggests that the regulations which have been proposed to curb microfinance clients' stress levels have ambiguous implications. Nevertheless, largely based on case-study evidence, the Indian federal and state governments have moved to increase regulation of the microfinance sector [14].

Here, we provide experimental evidence on a key product design feature – repayment frequency. Poor households' income is often irregular and uncertain [15]. As a result, frequent repayment requirements could be a source of stress. Yet, one can imagine *less* frequent repayment *increasing* financial stress if clients procrastinate in preparing and have to scramble to make a larger installment at the end of each month.

Observational evidence is unable to identify the causal impact of repayment flexibility on stress in large part because there is little variation in repayment schedules across microfinance clients, and because, where alternative payment plans are possible, clients who face differentially stressful economic lives are likely to select into the repayment schedule that best suits their needs. [16], for example, shows that microfinance clients who select into more flexible repayment schedules repay more of their loan. However, these results could be driven by either selection or a change in behavior due to the loan contracts. Here, we use a randomized experiment to provide causal evidence that more flexible repayment reduces client stress. Although we are unable to pinpoint the channel, the time trends in stress and income suggest that an important channel is likely to be the fact that flexible repayment schedules allow clients to invest in more profitable assets.

Our findings complement a growing experimental literature on the impact of microfinance. These studies report limited to no effects of the classic microfinance contract on average poverty rates among microfinance clients, despite significant benefits for some population subgroups [17]–[19]. Our study suggests that one reason for this may be that client investment behavior (and subsequent income) is sensitive to the design of microfinance debt contracts. In other words, if well designed, microfinance products have the potential to provide poor entrepreneurs with valuable credit that ultimately reduces their financial insecurity and related levels of stress, improving their economic and mental well-being.

## Materials and Methods

Any study that compares outcomes across microfinance clients who self-select into either the traditional weekly repayment schedule or the more flexible monthly repayment schedule potentially obfuscates the true impact of less frequent repayment on financial stress, since different types of clients are likely to sort into each repayment schedule. Our study addresses this concern by using a randomized controlled trial (RCT) experimental design. Harvard's Institutional Review Board approved the study design and protocol. Verbal informed consent was obtained from all participants (due to low education level among respondents, written consent requirement was waived).

We partnered with a large microfinance organization called Village Financial Society (VFS) in Kolkata, India. At the time of the study, VFS loans were distributed through five-member microfinance groups. Each client received an individual loan and her ability to obtain a subsequent loan depended only on her personal repayment record (individual liability as opposed to joint liability group lending). The loan was uncollateralized and the modal value was US $222 excluding interest costs, which were ten percent of the loan size. Clients were required to make periodic repayments to the loan officer beginning shortly after loan disbursal in a group meeting conducted in their neighborhoods.

In total, 213 clients participated in this study, all of whom were selected from a larger study group of 740 clients. Between January and September 2008, VFS recruited clients and formed 148 five-member groups comprising 740 clients. Loan sizes varied from Rs. 4000 to 12,000 (∼$90 to $260), with a modal loan size of Rs. 10,000. Randomization was implemented using a random sequence of numbers generated with statistical software by the project research assistant. Treatment status was assigned to batches of 20 groups at a time based on the timing of group formation with a 1∶1 allocation ratio.

After group formation and prior to loan disbursement, the field coordinator called the project research assistant to determine whether a group had been randomly assigned to either a five-weekly repayment schedule (from here on referred to as “monthly”) or a weekly repayment schedule. One exception is the first batch of treatment groups, which was composed of 12 groups assigned to a four-weekly repayment schedule as opposed to a five-weekly repayment schedule. The change to a five-weekly repayment schedule was made to better accommodate VFS' logistical needs. Clients on the four-weekly experiment were not selected for the study considered here. More information on client selection and randomization is available online in Text S3. Clients were informed that repayment would be determined by lottery. Since all members of a group were restricted to have the same repayment schedule, the trial was a parallel cluster-randomized trial.

From these 740 clients, we randomly invited 105 weekly and 105 monthly clients to participate in the Daily Consumption Survey (DCS). Selection was based on a random sequence of numbers generated with statistical software by the project research assistant. Due to a major festival scheduled to occur several weeks after the start of the DCS survey, we chose the monthly clients from the 21 monthly groups with starting dates that ensured that the DCS survey could run from one repayment to the next for monthly clients without interruption by major festivals. To ensure balance across treatment arms, we selected the weekly clients from the 74 weekly groups with group formation dates that overlapped with the 21 monthly groups.

Twenty-three of the 210 initial clients dropped out, including 11 from control and 12 from treatment. Although the attrition rates were similar for both groups, it is possible that the types of clients that dropped out of the treatment group were systematically different from those who dropped out of the control group. For this selection to generate the main results we present later, clients who dropped out of the control group would have to have significantly lower average baseline stress levels than clients dropping out of the treatment group, which is not supported by a comparison of baseline stress measures across attritors in both groups.

To maintain a target sample size of 200, we randomly selected an additional six weekly and seven monthly clients from the larger study group of 740. The sample size of 200 was chosen based on budget considerations. To summarize, a total of 111 weekly clients from 45 groups were randomly assigned and received the intended treatment, while 100 clients from 42 groups were analyzed for the primary outcomes. A total of 112 monthly clients from 26 groups were randomly assigned and received the intended treatment, while 100 clients from 26 groups were analyzed for the primary outcomes. In the next section, we discuss how we compute standard errors in light of the potential correlation of outcomes within loan groups.

On average, weekly clients paid US$5.40 every week, while monthly clients paid $27.10 every five weeks. The loan duration in both cases was 45 weeks.

In order to assess financial stress levels accurately and in real time, we employed an innovative application of cell phone technology to survey clients every 48 hours for seven weeks. Clients were surveyed on average 16.5 weeks after receiving the loan. By contacting the microfinance clients in our study via cell phones, which were provided to each client for the purpose of this study, we mitigated recall bias, reduced non-response and non-participation rates, and collected 5000 surveys (200 clients surveyed 25 times each) in a cost-effective manner. In order to truly understand consumption smoothing and liquidity constraints among the poor, one needs data that accurately measures consumption levels, income, and assets of households over time. Particularly for consumption data, several potential sources of reporting error have been documented in the economics literature, the most important of which are recall mistakes, inability to capture total household consumption, and level of aggregation of consumption categories [20], [21]. In our project, we have attempted to mitigate the risks posed by each while keeping logistical demands and costs of surveying reasonably low through a novel survey implementation strategy that leverages cell phone technology available in our study region. For more details on reporting error on consumption data, see [20].

Each time the survey was administered, we measured clients' level of financial stress with four questions: confidence in ability to repay loan, anxiety about loan repayment, argument with spouse about finances, and time spent thinking about repayment. We construct four indicator variables to capture financial stress: 1 if they did not feel confident about their ability to repay the loan; 1 if they felt worried, tense, or anxious about paying the next loan installment; 1 if they argued with their spouse in the last 24 hours; and 1 if they spent at least five minutes thinking about repayment during the past day. The Cronbach Alpha for these measures is high, at 0.8386, suggesting that it is appropriate to think of the different questions as measuring one underlying construct. Thus, in addition to the individual variables, we report the effect of the equally weighted average across the four outcomes. We call this construct the Financial Stress Index.

Self-reported financial stress is an important measure of household well-being. Indeed, in our sample, financial stress is positively correlated with observable indicators of poverty, although not at a statistically significant level. The average value of the Financial Stress Index is higher for clients who are illiterate, report not having a savings account, had a shock within the past 30 days, do not have a household business, and are in the lower half of the asset distribution.

Evidence on the health relevance of self-reported measures of stress comes from a large literature that documents significant correlation between stress biomarkers (which bear a direct relationship with human health) and self-reported measures [22]. conduct a literature review of studies documenting a correlation between blood pressure levels and self-reported measures of job strain. More similarly to the stress measure used here [23], find that responses to questions about ability to meet financial obligations, such as food, clothing and medical care, correlate with measures of blood pressure and cortisol response. Similarly [24], find herpes antibody levels and self-reported measures of stress are correlated in a sample of low-income women. However, the same study does not find an association between measures of salivary cortisol response and self-reported stress measures. The absence of a significant correlation between cortisol response and self-reported stress measures appears to hold more generally [25]. conducts a literature review of the correlation between salivary cortisol and self-reported mental stress measures and conclude, “the evaluation of the studies in this paper showed insufficient evidence for an association between self-reported mental stress and the cortisol response in field studies.” The authors also detail some of the difficulties in collecting saliva swabs in a reliable way for cortisol testing.

Because the contracts were randomly assigned to clients, a comparison between treatment arms has a causal interpretation. Ordinary Least Squares (OLS) regression analysis allows us to compare monthly and weekly clients controlling for variables such as day of the week, whether the survey took place in the morning, and cohort effects.

For all outcome variables we estimate simple ordinary least squares regressions of the following form: 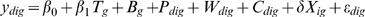 where *y_dig_* is the outcome of interest for client *i* in group *g* on day *d* and *T_g_* is an indicator variable that equals one if the group was assigned to the five-week repayment schedule. All regressions include dummies for stratification batch (*B_g_*), day of the week (*P_dig_*), whether the survey was taken in the morning (*M_dig_*), number of weeks since disbursement (*W_dig_*), and the calendar week (*C_dig_*). The vector *X_ig_*, which is present only in the specification labeled as including controls, consists of age, marital status, household size, Muslim, literate, has savings, negative shock in last month, has household business, total asset value, and loan size. Regressions including *X_ig_* also control for loan officer fixed effects. In all regressions, standard errors are corrected for clustering within loan groups using Huber-White standard errors.

Before turning to the results, we discuss our primary hypotheses regarding the possible channels of influence. Increased flexibility in repayment can influence mental stress among clients through several channels:

1. Income: Greater flexibility in repayment may allow clients to invest their microfinance loans in less liquid but more profitable business assets and inventory by providing more time between repayments to earn a return. As a hypothetical example, consider a small hardware store owner who knows that buying and selling higher-quality light fixtures yields higher profits. Yet, he cannot use his microfinance loan to buy this inventory because higher-quality light fixtures do not sell as quickly as lower-quality ones; hence, he will not have the money in time to make his first repayment if he invests in this more illiquid (but more profitable) inventory. If he repays every month instead of every week, he may be able to invest in the higher-quality inventory and increase profits. Since wealth is negatively correlated with financial stress, we should see higher income and reduced financial stress among clients with more flexible repayment schedules.
2. Self-control: Recent research has suggested that the poor may be more susceptible to temptation [26]. In such cases, less frequent repayment may increase stress and default. Specifically, less frequent repayment should increase both overall household expenditure and spending on “temptation goods” (for example, tobacco, alcohol, and ready-made foods).
3. Consumption smoothing: Much evidence indicates that avoiding large fluctuations in consumption is costly for the poor in developing countries [27]. Less frequent repayment can reduce the cost of smoothing consumption in the event of negative health and business shocks. This reduced cost to smoothing consumption may reduce variance of household expenditure and make it easier for the poor to meet their repayment obligations. Alternatively, if households would have used costly methods such as liquidating business assets or removing children from school to smooth consumption, it could manifest itself in higher income. In either case, we would expect a reduction in both stress and default.
4. Time burden: By reducing the number of repayment meetings clients are required to attend, a monthly repayment schedule relaxes the time constraint on clients by approximately 1.5 hours per month, which could reduce their overall stress levels.

We expect that these channels of influence will interact. For instance, less frequent repayment can increase client income while at the same time increasing default through its impact on fiscal discipline. Hence, the net effect of less frequent repayment on mental stress remains an empirical question.

## Results

**Figure 1:**
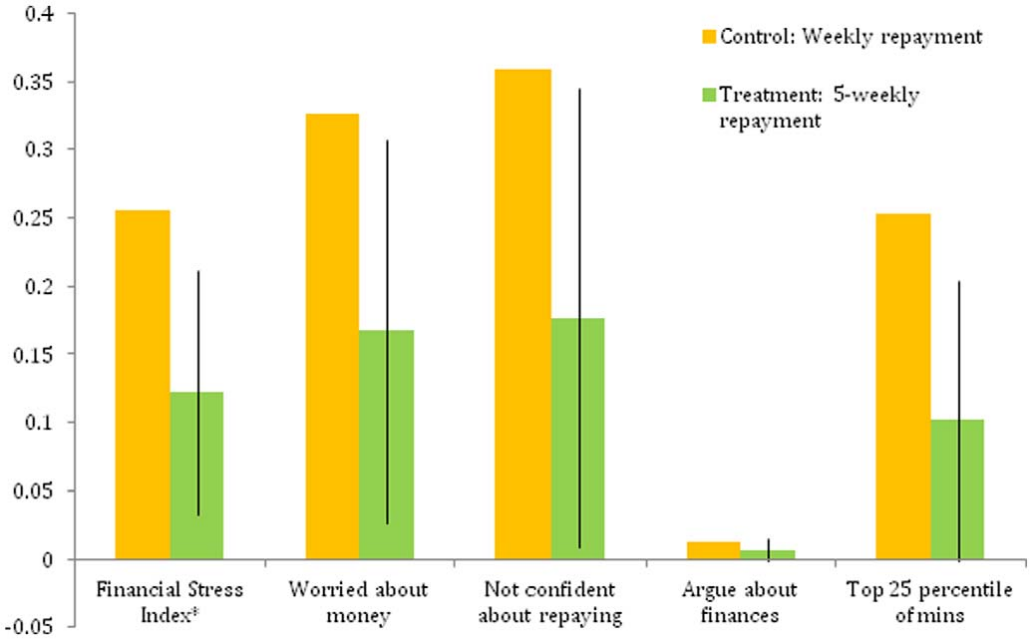
Impact of less frequent repayment on financial stress. Control bars represent means of control group. Treatment bars are sum of control group mean and treatment coefficient estimated by OLS regression. OLS regressions include control variables shown in Panel A of Table S1. Lines on Treatment bars represent plus or minus 1·96 times the standard error of the treatment coefficient. The Financial Stress Index is an unweighted average of “Worried about money,” “Not confident about repaying,” “Argue about finances,” and “Top 25 percentile of minutes spent.” Hence, while it can be represented on a 0 to 1 scale, it should not be interpreted as a percentage like its component measures.

Figure 1 shows that monthly clients scored 45 percent lower on the Financial Stress Index than weekly clients (P<0·05, *t test*). Monthly clients report being worried about repayment 51 percent less often than weekly clients (P<0·05, *t test*), and report a lack of confidence in their ability to repay their loan at a rate that is 54 percent lower than weekly clients (P<0·05, *t test*). Monthly clients are also 60 percent less likely to spend significant time thinking about loan repayment (P<0·05, *t test*).

The results show that flexibility in repayment reduced clients' mental stress along several dimensions, suggesting that product design can play a key role in influencing how microcredit affects the financial stress of the poor.

**Figure 2:**
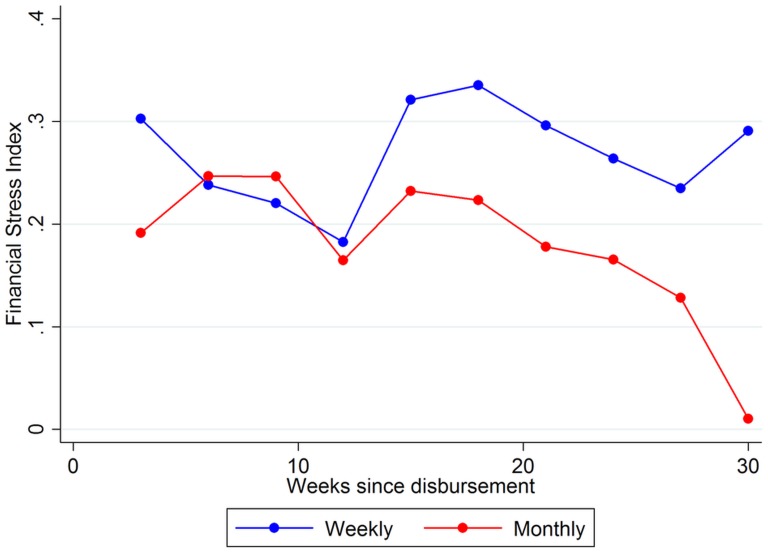
Time path of client financial stress. Dots are three-week averages of the Financial Stress Index, plotted separately for control and treatment groups.

**Figure 3:**
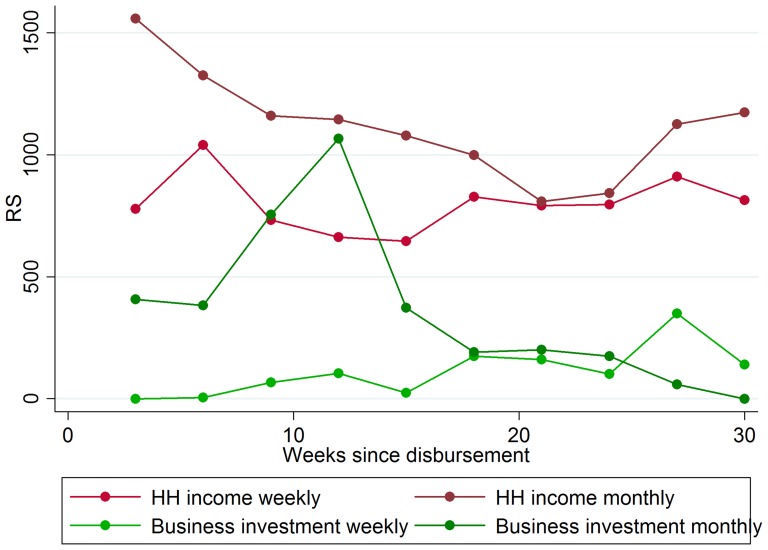
Time path of client income and business investment. Dots are three-week averages for household income and business investment, plotted separately for control and treatment groups.

Our survey provides suggestive evidence on the channels of influence associated with less frequent repayment:

1. Higher business income and household expenditures: Relative to weekly clients, monthly clients more than doubled their business income on average, increasing their total household income by 84–88 percent (P<0·05, *t test*), as shown in Table 1. As we would expect, wage income is unaffected by repayment frequency, reducing concerns that the result is spurious. Moreover, higher business profits among monthly clients were due to increased investment in business inventory. We note that the smaller sample size for the business investment and inventory outcome is due to the fact that we aggregate business investment and inventory to the client-week level. Finally, we also observe higher household expenditures, which is consistent with higher income. Considering that the correlation between financial deprivation and financial stress problems is well established in the literature, we hypothesize that this large increase in income is likely an important contributor to the financial stress results.
  Further evidence of the income channel comes from looking at the time path of stress levels and income. As shown in Figure 2, stress levels are more or less comparable between weekly and monthly clients until around week 12 of the loan cycle, at which point the stress level of monthly clients begins to fall steadily. The difference in stress levels between monthly and weekly clients is particularly large at the very end of the interview period (week 30), by which time large differences in income have also emerged (Figure 3). In contrast, we see that differences in investment are concentrated in the early part of the loan cycle (Figure 3). Given that income effects emerge slowly over time as investments come to fruition, if income is the driver of differences in financial stress, we would expect stress levels of monthly and weekly clients to diverge over time. The observed time path does not support the alternative possibilities that either consumption-smoothing or the time burden of repayment meetings are the channels; in those cases financial stress levels should converge as debt levels fall or remain constant throughout the loan cycle. The difference-in-differences estimate in column 2 of Table 1 confirms that the stress index is significantly lower for monthly clients after, but not before, week 12 of the loan cycle.
2. No increase in short-run default and share of spending on temptation goods: Using transaction data obtained from VFS, we tested whether moving to a monthly repayment schedule increased default by measuring default rates at 8, 16, and 20 weeks past the date when each client's loan was due in full. Using the OLS specification described above, we find no evidence that moving from weekly to monthly repayment increased default during the study period. Within our study period, household expenditures among monthly clients increased by only a fraction of the increase in household income, suggesting that clients were able to maintain fiscal discipline and prioritize business investment even with less frequent repayment requirements. The fraction of total household spending that was devoted to tobacco, alcohol and ready-made foods was unaffected.
3. No change in variation of income and consumption: We find no change in the coefficient of variation of income and household expenditure among monthly clients. It is possible that an improved ability to smooth consumption also increases economic income and the net effect is no change in the volatility of consumption [28]. As a result, this outcome cannot be interpreted as implying that monthly repayment failed to reduce the cost of smoothing consumption.

**Table 1:** Impact of less frequent repayment on financial stress and economic outcomes.

	Financial Stress Index	Financial Stress Index	Total income (RPS)	Business income (RPS)	Wage income (RPS)	Invest- ment in business inventory (RPS)	Default at 20 weeks past due date	Total house- hold expend- itures (RPS)	Share of expenditure on tobacco, alcohol, and foods prepared outside of home
	(1)	(2)	(3)	(4)	(5)	(6)	(7)	(8)	(9)
*Panel A (no controls)*									
Monthly	−0•093**	−0·0016	561·3**	614·9**	−53·59	523.7**	−0·0114	99·94**	0·00971
	(0·0442)	(0·0437)	(278·9)	(286·2)	(38·04)	(227·6)	(0·0115)	(47·62)	(0·0104)
Monthly X		−0119*							
post-12 week		(0·0616)							
*Panel B (controls)*									
Monthly	−0·134***	0·0592	503.0**	558.9**	−55.93	395.1	−0·0115	61.61	0·0146
	(0·0457)	(0·0573)	−278.9	−230.2	−39.88	−249.6	(0·0115)	−53.29	(0·0109)
Monthly X		−0·242***							
post-12 week		(0·0673)							
Observations	4,928	4,928	4,999	4,999	4,999	1,599	200	4,999	4894
Clients	200	200	200	200	200	200	200	200	200
Clusters	68	68	68	68	68	68	68	68	68
Intra-cluster corr.	0.309	1.309	0.239	0.266	0.0271	0.151	0.174	0.0414	0.0828
Mean for weekly	0·256	0·257	634·1	469·3	164·9	477·1	0·01	414·1	0·0793

Intent-to-treat effects of monthly repayment schedules on outcomes. Table shows OLS results for the independent variable “monthly”; Huber-White SEs and control group means are also shown. Regression in column 2 includes two additional independent variables: post-12 week dummy which is an indicator variable which equals one if survey occurred more than 12 weeks after disbursement (not reported) and the interaction of monthly and post-12 week dummy (reported).Variation in sample sizes is due to survey non-response. The Financial Stress Index, as described in Figure 1, is an unweighted average of “Worried about money,” “Not confident about repaying,” “Argue about finances,” and “Top 25 percentile of minutes spent.” Estimates where controls are used include variables listed in Panel A of Table S1.

* *p<0·10,*

** *p<0·05,*

*** *p<0·01.*

## Discussion

Presented with the problems caused by financial stress, policymakers often believe the right response to is to reduce overall financial indebtedness. But there are many reasons to believe that access to credit is critical to improving economic outcomes for the poor in developing countries. In this study we consider self-reported measures, which allow us to ask specifically about the stress that arises from managing money or finding the means to pay for the next loan installment. Our results show that, holding availability of credit constant, changing the terms of the contract can significantly alter these stress measures. In particular, increasing repayment flexibility greatly reduces the mental health burden of indebtedness.

We find little evidence that the less frequent payments affected social interactions, default, spending on temptation goods, or clients' ability to smooth income.

Rather, our results suggest that a schedule requiring less frequent payments leads to a reduction in financial stress because it enables clients to use their credit more wisely and take advantage of profitable investment opportunities, which results in higher household income.

## Supporting Information

Table S1 **Weekly vs. 5-weekly Randomization Check.**
(XLS)

Table S2 **Coefficient of Variation.**
(XLS)

Text S1 **Description of Village Financial Society.**
(DOC)

Text S2 **Data Collection.**
(DOC)

Text S3 **Statistical Methodology.**
(DOC)

Text S4 **Stress Questions.**
(DOC)
