# Supplementary material for: Repayment Flexibility Can Reduce Financial Stress: A Randomized Control Trial with Microfinance Clients in India
Source: PLoS One. 2012 Sep 26;7(9):e45679. doi: 10.1371/journal.pone.0045679 (PMC3458929; doi:10.1371/journal.pone.0045679)
Supplement: Text S1 — Description of Village Financial Society. (DOC) [file pone.0045679.s003.doc]

Description of Village Financial Society

Our project was implemented with our partner MFI, the Village Financial Services (VFS), which started operations in the Indian state of West Bengal in 1982. It is larger than the typical MFI in India, with nearly 60 offices, total assets of 30.1 million USD, and 184,000 active borrowers as of 2009, compared to the median Indian MFI which has 27 offices, 10.7 million USD and 65,000 active borrowers.

In light of the current debate on the impact of MFIs on clients’ welfare in India, additional statistics on VFS’s finances and operations may be of interest. Compared to a median Indian MFI, VFS clients carry a lower loan balance with the average loan balance per borrower/ GNI per capita at 12% for VFS versus 14% for the median Indian MFI. VFS also achieves a lower return on assets and return on equity, 1.1% and 7.1% respectively, compared to 1.8% and 10.5% for the median Indian MFI. VFS has a borrower-to-staff member ratio of 338, which is greater than the 75^th^ percentile for all Indian MFIs, and their cost per borrower, at 15 USD is equal to the median. The percentage of their portfolio at risk greater than 90 days is 0.54% compared to the median of 0.33% for all Indian MFIs (MIX Market, 2011).

Despite being in an urban environment, VFS clients seem to have limited outside borrowing. In our baseline survey, only 6.2% of our entire sample report having taken out a non-VFS loan in the past two years.
