# Supplementary material for: Repayment Flexibility Can Reduce Financial Stress: A Randomized Control Trial with Microfinance Clients in India
Source: PLoS One. 2012 Sep 26;7(9):e45679. doi: 10.1371/journal.pone.0045679 (PMC3458929; doi:10.1371/journal.pone.0045679)
Supplement: Text S2 — Data Collection. (DOC) [file pone.0045679.s004.doc]

Data Collection

In order to truly understand consumption smoothing and liquidity constraints among the poor, one needs data that accurately measures consumption levels, income, and assets of households over time. Particularly for consumption data, several potential sources of reporting error have been documented in the Economics literature, the most important of which are recall mistakes, inability to capture total household consumption, and level of aggregation of consumption categories (Beegle et al., 2010). In our project, we have attempted to mitigate the risks posed by each while keeping logistical demands and costs of surveying reasonably low through a novel survey implementation strategy that leverages cell phone technology available in our study region.

Firstly, recall error, or the misreporting of true consumption by the respondent over the period of recall due to faulty memory, has been documented by several studies to be a major source of bias in consumption survey data. More specifically, longer recall periods tend to be associated with greater underreporting of consumption. For example, the experiments conducted by Scott and Amenuvegbe (1990) with households from the Ghanaian Living Standards Survey concluded that reported expenditures fell at an average of 2.9 percent for every day added to the recall period. Since the focus of our study is not only the average level of consumption but also its fluctuation over time, high-frequency data is even more critical to ensure accurate and precise measurements. As Samphantharak and Townsend (2010) write, “high-frequency data is necessary for the analysis of liquidity, the short-term smoothing of consumption, the protection of investment from cash flow fluctuations, and the financing of cash flow budget deficits.”

A second important source of error is the inability of the survey respondent to account for total household consumption. Individual consumption, particularly by other adult members of the household, may occur outside the knowledge of the survey respondent and may not be captured in the consumption survey. For example, Beegle et al. (2010) compare several different methods for collecting consumption data among households in Tanzania using a randomized experimental design. In one treatment arm, each household was given a household consumption diary while in another treatment arm, each adult member of each household was given a personal consumption diary. The first treatment arm produced mean consumption measures that were 19 percent lower than the second, providing evidence that the traditional approach of relying on one adult respondent underestimates consumption activities for the household. Similar results were found in experiments with personal and household consumption diaries in Russia (World Bank, 2005).

The last source of potential error is the level of aggregation of consumption categories. On one hand, it is necessary to disaggregate consumption to some extent to get reasonably accurate responses about total expenditure levels (Deaton, 1997). Moreover, there is evidence that reducing the number of categories into which consumption is disaggregated lowers total expenditure reported (Deaton, 1997). However, longer questionnaires often entail higher survey costs, more time, and risk of greater non-response and survey attrition.

Due to the high cost and logistical complexity of collecting accurate and detailed consumption data on a real-time basis, empirical data on day-to-day consumption patterns of the poor in developing countries have been limited. Previous studies have largely relied on questions that risk the noise and bias of imperfect recall, often over long reference periods (e.g., “how many times have you run out of food in the last year?”) or on datasets that provide detailed consumption data but from a very small sample size over short time periods.

A major innovation of our project is to produce a dataset that contains detailed information about daily expenditures and consumption from 200 Indian households over 50 days using cell phone technology. We call this effort the Daily Consumption Survey (DCS) project. The DCS survey was administered to each client via cell phone every other day for a total of 25 times, covering a time period of 50 days in total. Each DCS participant was offered the option of taking two pre-purchased CDMA phones, one for the client and one for her husband, locked with a pre-determined service provider. In total, 350 phones were distributed, 200 to respondents and 150 to husbands. Air time for surveys was pre-paid by the experiment. To provide incentives to participate in the survey and to ensure that the phones were returned at the end of the survey period, participating clients were offered Rs. 5 for every call answered by them or their husband, deliverable upon the return of the cell phones at the end of the survey period.

Each time a client was surveyed, she was asked questions about her own earnings, transfers received and sent, and loans. She was also asked about the household’s expenditure on food, housing, education, healthcare, loan repayment, and savings installments. Lastly, she was asked about the time she spent working and her level of mental stress related to loan repayment and finances. Each time a client was surveyed, her husband was also surveyed and asked about his earnings, transfers received and sent, and loan payments. He was also asked about time spent working and his level of mental stress related to loan repayment and finances. Each administration of the survey took on average 12 to 13 minutes and 90% of surveys took less than 25 minutes. Response rates were very high, with only two survey administrations missing from the 5000 total surveys that were collected (200 clients with 25 surveys each).

In addition to contributing a unique dataset to the economic development field, the DCS addresses many of the potential sources of bias in the collection of consumption data outlined above. First, by contacting the respondent household every 48 hours, we capture a nearly real-time view of consumption activity of the household, mitigating the potential for recall bias. Second, our data collection strategy used enumerators to collect data instead of relying on self-reporting in consumption dairies, in which the frequency and consistency of reporting is difficult or impossible to monitor. By making use of cell phone technology, we were able to employ a relatively costly approach (enumerators) in a cost-effective manner. And third, this approach helped cut down on non-response and non-participation because it was also lower cost for clients to participate relative to the hassle and recall problems of having to fill out a daily consumption diary.

Fourth, this method allowed us to interview multiple members of the household without having to return to the household at multiple times during the day, which gave us the possibility of surveying both husbands and wives without enormous added costs of selection of participating households. This aspect of our consumption survey is another major advantage to these data. That is, in addition to surveying the microfinance client, we also interviewed her husband for each survey, asking complementary but non-overlapping questions that would help produce a more complete picture of household consumption. Lastly, we balanced the need to get more detailed consumption data from respondents with the concern of survey fatigue by choosing 62 consumption categories to be included in the survey for clients (excluding questions about income or business investment).
