# Supplementary material for: Repayment Flexibility Can Reduce Financial Stress: A Randomized Control Trial with Microfinance Clients in India
Source: PLoS One. 2012 Sep 26;7(9):e45679. doi: 10.1371/journal.pone.0045679 (PMC3458929; doi:10.1371/journal.pone.0045679)
Supplement: Text S3 — Statistical Methodology. (DOC) [file pone.0045679.s005.doc]

Statistical Methodology

Between January and September 2008, VFS recruited clients and formed 148 five-member groups comprising of 740 clients. Loan sizes varied from Rs. 4000 to 12000 (~$90 to $260), with a modal loan size of Rs. 10000. Randomization was implemented using a random sequence of numbers generated with statistical software by the project research assistant. Treatment status was assigned to batches of 20 groups at a time based on the timing of group formation with a 1:1 allocation ratio. One exception is the first batch of treatment groups, which was comprised of 12 groups assigned to a four-weekly repayment schedule as opposed to a five-weekly repayment schedule. The change to a five-weekly repayment schedule was made to better accommodate VFS’ logistical needs. For all 740 clients, we conducted baseline and endline surveys, which contain information about consumption, household shocks, housing, assets and income, transfers, employment, business activity, and women’s empowerment, among other topics.

Clients assigned to four or five-weekly repayment schedules met and repaid their loans every four or five weeks, while clients assigned to the weekly repayment followed the traditional schedule of meeting and repaying every week. Other features of the loan contract were held constant across the two groups, including interest rates at 12%. Both groups had 45 weeks from the time of the loan disbursement to repay their loans in full. For the majority (nearly 90%) of clients in the DCS dataset, the first DCS survey was given at least 45 days after the loan disbursement.

After group formation and prior to loan disbursement, the field coordinator called the project research assistant to determine whether a group had been randomly assigned to either a five-weekly repayment schedule (from here on referred to as “monthly”) or a weekly repayment schedule. Since all members of a group were restricted to have the same repayment schedule, the trial was a parallel cluster-randomized trial.

Of the 740 clients participating in the larger VFS experiment, we selected 105 five-week clients and 105 weekly clients and invited them to participate in the DCS project. Due to a major festival scheduled to occur several weeks after the start of the DCS survey (Durga Puja), we had to choose the five-week clients from among the 21 five-week groups whose starting date would ensure that the DCS survey could run from one repayment to the next without interruption of this festival. We randomly selected 105 clients from the 370 weekly clients in the larger experiment, resulting in 42 of the 74 weekly groups being represented in DCS. Twenty-three of the 210 initial clients dropped out, 11 from control and 12 from treatment; hence, there is no differential attrition between weekly and five-week clients in terms of number of respondents. We randomly sampled from the remaining clients in the larger experiment to replace six clients in control and seven clients in treatment for a final total of 200 clients participating in the DCS survey. We compare clients who left the original sample versus those who make up the final sample along a range of traits — discount rates (measure of patience), income, and employment status (table available upon request). We find no evidence that those in the final sample are more patient than those who attritted, mitigating one potential concern regarding the external validity of our results. We do find evidence that suggests that those who attritted from the sample were slightly less likely to be working in the past week (90 percent versus 98 percent).
