# Supplementary material for: Repayment Flexibility Can Reduce Financial Stress: A Randomized Control Trial with Microfinance Clients in India
Source: PLoS One. 2012 Sep 26;7(9):e45679. doi: 10.1371/journal.pone.0045679 (PMC3458929; doi:10.1371/journal.pone.0045679)
Supplement: Text S4 — Stress Questions. (DOC) [file pone.0045679.s006.doc]

Stress Questions

Each time the survey was administered, we measured clients’ level of financial stress with four questions:

1. `` How Confident do you feel that you will be able to repay next loan installment”. We allowed for responses on a 4 point scale, where 1 was confident and 2-4 expressed increasing degree of non-confidence. We collapse the responses into an indicator variable which equals one if the client response took values 2-4.
2. `` Do you currently feel worried, tense or anxious about paying next loan installment”. We allowed for responses on a three point scale, ranging from worried (1) to not at all (3). We collapse the responses to an indicator variable which equals one if client reported feeling slightly or very worried (response values 1-2)
3. ``How many minutes do you spend thinking about loan?” We allowed for continuous range of responses (in minutes)
4. ``Do you argue with your spouse about finances?” We allowed for discrete yes, no response.
